# The complete chloroplast genome of the desert shrub *Nitraria sphaerocarpa* (Nitrariaceae) and phylogenetic analysis

**DOI:** 10.1080/23802359.2021.1934580

**Published:** 2021-06-14

**Authors:** Feng Song, Ying Feng

**Affiliations:** a State Key Laboratory of Desert and Oasis Ecology, Xinjiang Institute of Ecology and Geography, Chinese Academy of Sciences, Urumqi, China; b University of Chinese Academy of Sciences, Beijing, China

**Keywords:** Chloroplast genome, Nitrariaceae, *Nitraria sphaerocarpa*, phylogenetic analysis

## Abstract

*Nitraria sphaerocarpa* Maxim. is a typical desert shrub commonly used as a sand binder. Here, we sequenced and characterized the whole plastid genome of *N. sphaerocarpa*. It is 159,369 bp in length, containing two copies of inverted repeat (IR) regions (26,566 bp, each), a large single-copy (LSC) region (87,854 bp), and a small single-copy (SSC) region (18,383 bp). It has 114 unigenes, including 79 protein-coding genes, 30 tRNA genes, four rRNA genes, and one pseudogene (*infA*). Phylogenetic analysis shows that *N. sphaerocarpa* is located at a basal position of the genus *Nitraria*.

*Nitraria sphaerocarpa* Maxim. (Nitrariaceae) is a deciduous and drought-tolerant shrub. It occurs in deserts, foothills, gravelly and sandy areas of north-western China. Its sterile branches have spiny at apex, leathery leaves, and white flowers. When the fruit is ripe, the exocarp becomes membranous and expand into a ball, a unique morphological feature of *N. sphaerocarpa* that differs from the other four species of *Nitraria* in China (Liu and Zhou 2008). *Nitraria sphaerocarpa* is resistant to drought and sand. It is commonly used to stabilize sand dunes in north-western China. There are around nine species in the genus *Nitraria* from Western Sahara to Central Asia and Australia. We acquired the complete chloroplast (cp) genome by genome skimming approach, which provides resources for studying the phylogenetic evolution and speciation of this taxon.

In this study, the sample of *N. sphaerocarpa* was collected from Heshuo, Xinjiang Uygur Autonomous Region (42°13′33.94″N, 87°20′49.59″E). The voucher specimen was deposited in the Herbarium of the Xinjiang Institute of Ecology and Geography, Chinese Academy of Sciences (XJBI, Ying Feng, luckfy@ms.xjb.ac.cn), with a collection number of NL-1-6. Total genomic DNA was extracted from approximately 100 mg of silica-dried leaves material with a modified CTAB method (Doyle 1987). DNA extracts were fragmented for 300 bp short-insert library construction and sequenced 2 × 150 bp paired-end reads on an Illumina HiSeq X-Ten instrument at Beijing Genomics Institute (Shenzhen, China). The filtering of raw reads uses Trimmomatic 0.35 (Bolger et al. 2014) to remove adapters and low-quality bases. Then, about 3.0 GB clean reads were assembled using GetOrganelle (Jin et al. 2018). The finished cp genomes were annotated with GeSeq (Tillich et al. 2017) and adjusted manually using Geneious v 11.0.2 (Ripma et al. 2014), with the reference genome of *N. tangutorum* (MH457633). Finally, the annotated cp genomes were submitted to GenBank (Accession number: MW820161). MISA-web v2.1 (Beier et al. 2017) was applied to identify simple sequence repeats sequences (SSR).

The whole cp genome of *N. sphaerocarpa* is 159,369 bp in size (37.3% GC contents), with a typical quadripartite genome organization, including a large-single copy (LSC, 87,854 bp, 35.2% GC contents) region, a small-single copy (SSC, 18,383 bp, 31.4% GC contents) region, and a pair of two inverted repeats (IR, 26,566 bp, 42.7% GC contents) regions. A total of 80 SSRs were detected, 75 of which were mono-nucleotide (A/C/T, 93.75%), four were di-nucleotides (AT/TA, 5.0%), one was tri-nucleotides (AAT, 1.25%), respectively. The cp genome encoded 114 unigenes, including 79 protein-coding genes, 30 tRNA genes, four rRNA genes, and one pseudogene (*infA*). Among these genes, 14 genes contained one intron and three contained two introns (*rps12*, *clpP*, and *ycf3*).

To investigate the phylogenetic position of *N. sphaerocarpa*, we performed a phylogenetic analysis based on complete cp genomes of five Nitrariaceae species and six species representing other 6 families within Sapindales. One species of Malvales was chosen as an outgroup. The DNA sequences for these 12 complete chloroplast genomes (after removing one IR) were aligned using the default option implemented in MAFFT version 7 (Katoh and Standley 2013). The most appropriate model (GTR + I + G) was determined by jModeltest v 2.1.10 (Darriba et al. 2012), and then Maximum likelihood (ML) tree was generated in RAxML 8.2.10 (Stamatakis 2014) with 1000 replicates (Figure 1). The phylogenetic tree showed that Nitrariaceae is in a basal position among the seven families of Sapindales. Consistent with Zhang et al. (2015), *N. sphaerocarpa* was located at a basal position of this genus. Besides, to find hyper-variable regions among four species of *Nitraria*, the sliding window analysis was performed by DnaSP v6 (Rozas et al. 2017), with a 1000 bp window and a 600 bp step size. Four hyper-variable regions (Pi > 0.004) in these genomes were identified, two of which are intergenic regions (*trnH^GUG^-psbA* and *rpoB-trnC^GCA^*, located in the LSC region), and two are protein-coding regions (*ndhF* and *ycf1*, located in the SSC and IR region, respectively). These hyper-variable regions can be great potential as taxon-specific barcodes for species identification within *Nitraria* genus.

**Figure 1.**
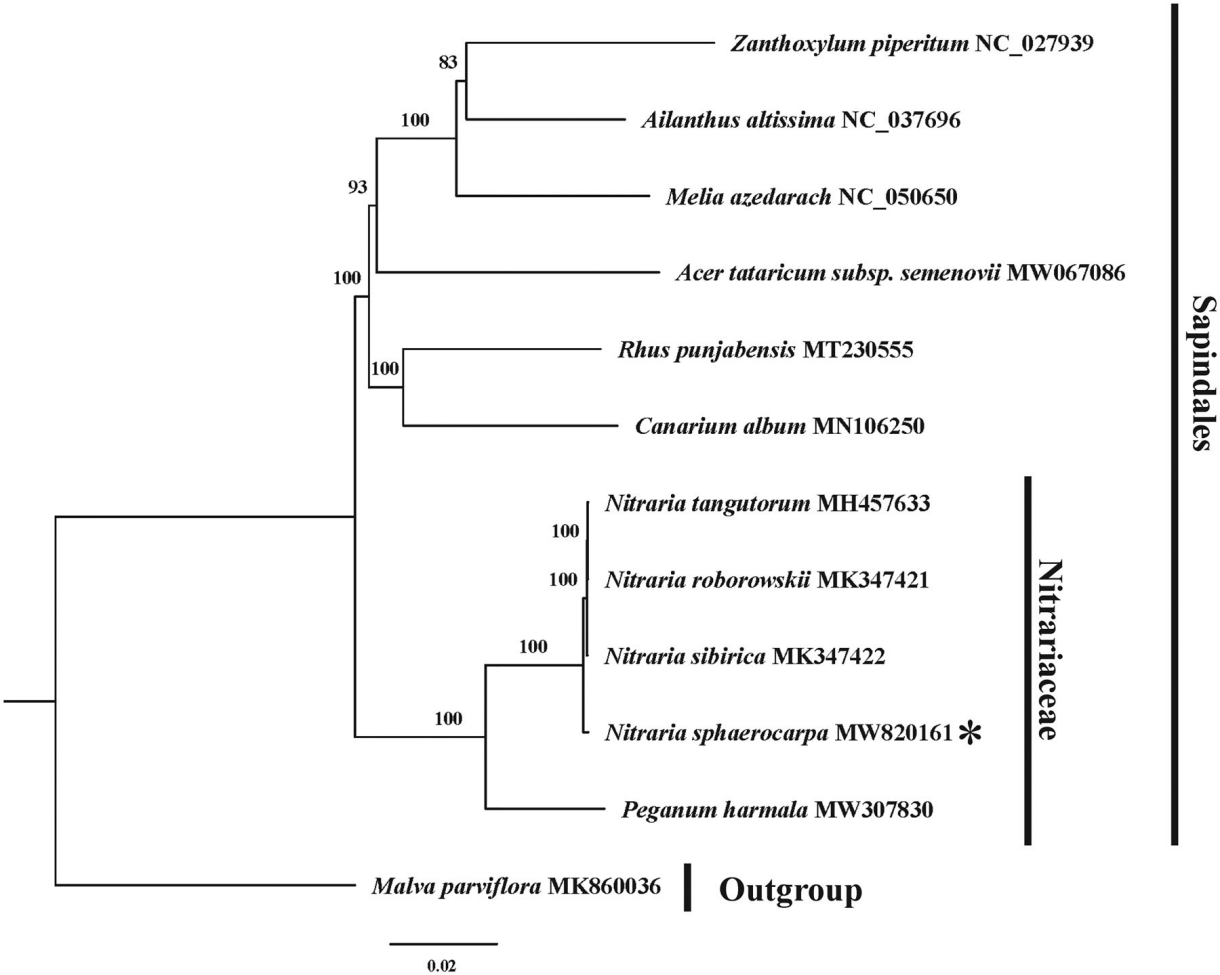
The maximum-likelihood (ML) phylogenetic tree of 12 species (11 of Sapindales and one from Malvales was chosen as outgroup) based on complete chloroplast genomes (only one IR region). The number above branches are bootstrap support values.

## Data Availability

The data that support the findings of this study are openly available in GenBank at https://www.ncbi.nlm.nih.gov/genbank/, accession number: MW820161. The raw sequencing data is available under GenBank Bioproject PRJNA728758.
